# Health system strengthening in fragile and conflict-affected states: a call to action

**DOI:** 10.1186/s12913-021-06753-1

**Published:** 2021-07-23

**Authors:** Michel D. Landry, Clarissa Giebel, Tillie L. Cryer

**Affiliations:** 1 Duke University, Durham, NC USA; 2 University of Liverpool & NIHR ARC NWC, Liverpool, UK; 3 BMC, part of Springer Nature, London, UK

## Abstract

Although the speed of global development has been impressive, not all countries have developed at the same pace. The World Bank Group (WBG) report that Fragile and Conflict-Affected States (FCAS) are those countries that have lower health and development outcomes, and risk not being able to achieve Sustainable Development Goals (SDGs) in the next few years. Health systems play an important role in building capacity and infrastructure that can lead towards fulfilling the SDGs. In this editorial, we set the context, and launch a call to action, for a *BMC Health Service Research* Collection titled “Health services and systems in fragile and conflict-affected regions”.

## Main text

On a global scale, progress has been made, and there have been impressive advances in health, development, and technology. For example, the World Health Organization (WHO) reports that global Under-5 mortality rates have dropped by close to 60% over the last 3 decades [1], and other health system-related indicators have followed similar positive trends. However, after 25 years of steadily declining extreme poverty rates, the World Bank Group (WBG) reported that extreme poverty around the world rose in 2020 [2]. They warn that the projected direct and indirect effects from the COVID-19 pandemic may further aggravate this worrisome trend. It may be rather self-evident, but worth restating in the context of this call to action, that the pandemic has highlighted the structural differences, both across and within different health systems.

Despite important advances, we as a global community remain far from reaching equitable and adequate health and social care outcomes that are required to achieve the United Nations (UN) Sustainable Development Goal 3 (SDG3) of ‘good health and wellbeing’ for all [3]. For instance, the UN estimates that less than half of the global population is covered or insured to receive essential health services [4]. Many of the most vulnerable parts of the global population reside in low and middle-income countries (LMICs) where services are often limited and frequently costly, leaving many with poor physical and mental health status. Other factors such as climate change, rising inequalities, and widespread corruption breed social unrest that often spirals vulnerable populations into precarious situations.

**Fig. 1:**
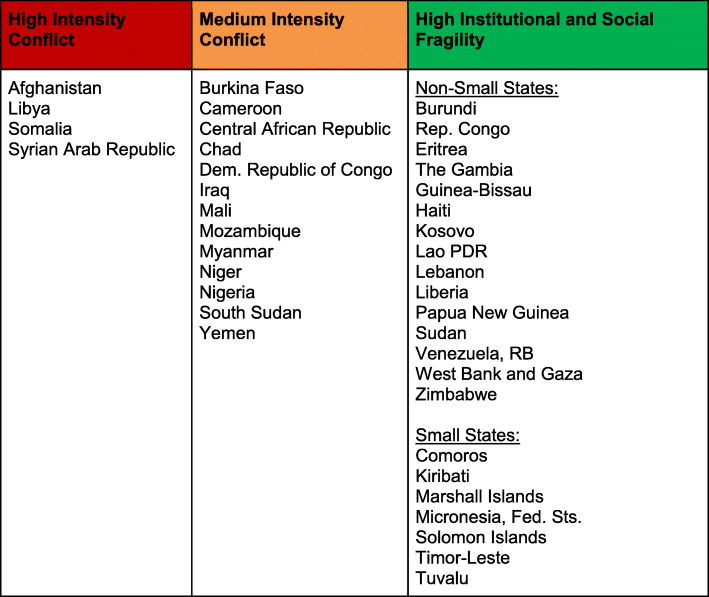
Fragile and Conflict Affected States (FCAS), 2020 [5, 6]

Beginning in 2006, WBG began to identify countries that were particularly vulnerable from a system perspective and began to openly publish a list of countries that have become known as Fragile and Conflict Affected-States (FCAS) [5, 6]. As indicated by Fig. 1, the vast majority of FCAS are located in Africa or the Middle East, and most are also categorized as low and middle-income countries (LMICs). These represent some of the most unstable nations, and it may be noteworthy that up to two-thirds of the world’s population who live in extreme poverty, defined as living on less than $1.90 US dollars per day, also live in FCAS. Even more worrisome is that the WBG estimates that by 2030, up to two-thirds of the world’s extreme poor could live in FCAS [7]. Moreover, Witter suggested that high levels of health and development needs within FCAS pose a significant threat or risk to the international community [7].

The WBG acknowledges that the list of FCAS is not comprehensive, and reports that the list “… functions primarily as a tool to help the WBG adapt its approaches, policies, and instruments in difficult and complex environments” [5]. That said, the criteria used to construct the annual list of FCAS include variables such as high levels of institutional and social fragility and violent conflict, and a relatively low ranking on the harmonized Country Policy and Institutional Assessment (CPIS) which is a composite scoring of the extent to which a country’s policy and institutional frameworks are aligned to poverty reduction, sustainable growth and the effectiveness of overseas development assistance. Additionally, criteria used to assess countries include the presence of the UN Department of Peace Operations (DPO) in the country and evidence of forced migration of 200 people (or more) per 100,000 population. Overall, FCAS are those nations that are experiencing low levels of economic development and experiencing protracted conflict or high levels of population mobility that have rendered the country and its population vulnerable and at risk.

Regardless of how the constructs of ‘fragility’ and ‘conflict’ are characterized, we suggest that health services and systems, and the ability to access health care, have a consequential impact on populations living in FCAS. However, when resources are already scarce, basic necessities including food, water, and shelter often become a priority leaving health and health services as a secondary consideration. The consequences of the COVID-19 pandemic have, and will, exacerbate these difficulties showcasing how health has and might further be neglected as a building block to reaching SDG3 within FCAS.

In response to these scarcity challenges that persist in austere settings, health systems in LMICs are often forced to be innovative in care delivery and financing. As a case in point, adequate, easily available and affordable mental health care is an essential service but is often neglected in LMICs [8]. Thus, mental health services are often left to community organizations and initiatives that try and address mental health, if at all [9]. COVID-19 has caused particular mental health and well-being concerns across the globe. There is significant evidence that mental health in pre-pandemic times was not well tended to in LMICs, including FCAS, with COVID-19 likely further exacerbating existing problems. Some of our research has shown the negative emotional impact on older adults living in Uganda during the pandemic for example [10]. However, important gaps in knowledge remain in the mental health services literature, including redesigning policies and frameworks, implementing health and mental health services, and the delivery of holistic, sensitive, and good quality care that adequately meets individual needs.

At the broader health system level, Noman et al. [11] provided evidence of challenging outcomes when international workforce regulations are not fully implemented in FCAS. Nevertheless, over the years, there have been advances in guidelines, frameworks, and structures to improve the delivery of health services in emergencies or humanitarian crises. For instance, Ansbro et al. [12] recently suggested the Reach, Effectiveness, Adoption and acceptance, Implementation and Maintenance (RE-AIM) implementation framework can be a valuable tool for evaluating complex interventions in humanitarian crisis settings.

The significant challenges faced in FCAS surrounding health system strengthening (HSS) are an urgent ‘call to action’ to consider ways in which to optimize and sustainably implement adequate health systems in FCAS across the globe. Action is needed if we are to meet SDG3 by 2030, less than 9 years from the writing of this editorial. In response to this, *BMC Health Services Research* calls upon the global health services research community to consider how we can sustainably strengthen health systems and services in FCAS. We are now welcoming submissions to our new collection, **Health services and systems in fragile and conflict-affected regions**. More details can be located here: https://www.biomedcentral.com/collections/HSSFCAR. We invite researchers, particularly those living in FCAS and LMIC, to consider submitting their work. We hope that this collection will provide a platform for shared learning about how we can strengthen health systems in FCAS and showcase creative ways to improve the health and wellbeing of those living in FCAS. We are particularly interested in submissions from FCAS, but we also encourage research from non-FCAS that have accepted important numbers of refugee populations and asylum seekers.

In order to reach SDG3 by 2030, the world will need to ensure that all countries, and under all scenarios, strive to reach the goal of ‘good health and wellbeing’ for all. We all have a responsibility to each other in this objective at the micro, meso, and macro levels, and we must ensure that ‘no one is left behind.’

## Data Availability

Data sharing is not applicable to this article as no datasets were generated or analyzed.

## Abbreviations

WBG: World bank group
FCAS: Fragile and conflict-affected states
SDGs: Sustainable development goals
WHO: World health organization
UN: United Nations
LMICs: Low and middle-income countries
CPIS: Country policy and institutional assessment
DPO: UN department of peace operations
RE-AIM: Reach, effectiveness, adoption and acceptance, Implementation and Maintenance implementation framework
HSS: Health system strengthening

## References

[1] World Health Organization (WHO). Children: Improving survival and well-being. Available from URL: https://www.who.int/news-room/fact-sheets/detail/children-reducing-mortality. Accessed on June 29, 2021.

[2] The World Bank. Poverty. Available from URL: https://www.worldbank.org/en/topic/poverty/overview. Accessed on June 29, 2021.

[3] The United Nations High Commission for Refugees (UNHCR). The Sustainable Development Goals and the Global Compact on Refugees Working together to ensure that refugees and host communities are not left behind. Available from URL: https://www.unhcr.org/5efcb5004.pdf. Accessed on June 30, 2021.

[4] United Nations. The sustainable development goals report 2020. Available from https://unstats.un.org/sdgs/report/2020/. Accessed on June 29, 2021.

[5] The World Bank. Fragility, Conflict & Violence. Available from URL: https://www.worldbank.org/en/topic/fragilityconflictviolence/overview. Accessed on June 29, 2021.

[6] The World Bank. Classification of Fragile and Conflict-Affected Situations. Available from URL: https://www.worldbank.org/en/topic/fragilityconflictviolence/brief/harmonized-list-of-fragile-situations. Accessed on June 29, 2021.

[7] Witter S (2012). Health financing in fragile and post-conflict states: what do we know and what are the gaps?. Soc Sci Med.

[8] Semrau M, Alem A, Ayuso-Mateos J, Chisholm D, Gureje O, Hanlon C, Thornicroft G (2019). Strengthening mental health systems in low- and middle-income countries: recommendations from the emerald programme. BJPsych Open.

[9] Kohrt, B.A.; Asher, L.; Bhardwaj, A.; Fazel, M.; Jordans, M.J.D.; Mutamba, B.B.; Nadkarni, A.; Pedersen, G.A.; Singla, D.R.; Patel, V. The role of communities in mental health Care in low- and Middle-Income Countries: a meta-review of components and competencies. Int J Environ Res Public Health 2018, 15, 1279. https://doi.org/10.3390/ijerph15061279, 6. 10.3390/ijerph15061279 PMC6025474 29914185

[10] Giebel C, Ivan B, Ddumba I. (2021) COVID-19 public health restrictions and older adults’ well-being in Uganda: psychological impacts and coping mechanisms, clinical gerontologist 2021, DOI: 10.1080/07317115.2021.1910394, 1, 9 10.1080/07317115.2021.1910394 33843497

[11] Noman, H., Dureab, F., Ahmed, I., al Serouri A., Hussein T., Jahn A. Mind the gap: an analysis of core capacities of the international health regulations (2005) to respond to outbreaks in Yemen. BMC Health Serv Res 21**,** 477 (2021). https://doi.org/10.1186/s12913-021-06395-3, 1 10.1186/s12913-021-06395-3 PMC8134964 34016124

[12] Ansbro, É., Homan, T., Qasem, J., Bil K., Rasoul Tarawneh M., Roberts B., Perel P., Jobanputra K. MSF experiences providing multidisciplinary primary level NCD care for Syrian refugees and the host population in Jordan: an implementation study guided by the RE-AIM framework. BMC Health Serv Res 21**,** 381 (2021). https://doi.org/10.1186/s12913-021-06333-3, 1. 10.1186/s12913-021-06333-3 PMC8074194 33896418

